# Intrauterine ovarian torsion with autoamputation and intra-abdominal wandering mass: a report of three cases and literature review

**DOI:** 10.3389/fmed.2025.1509477

**Published:** 2025-04-16

**Authors:** Xinxing Liu, Guojian Ding, Wenchao Tian, Xijie Liu, Tingliang Fu, Hongzhen Liu, Aiqun Xu, Xinling Han, Wenyu Feng, Lei Geng

**Affiliations:** ^1^Department of Pediatric Surgery, Binzhou Medical University Hospital, Binzhou, Shandong, China; ^2^Department of Pediatric Surgery, Children’s Hospital Affiliated to Shandong University, Jinan, China; ^3^Department of Gynaecology and Obstetrics, Yantai Affiliated Hospital of Binzhou Medical University, Yantai, Shandong, China; ^4^Department of General Surgery, Caoxian People’s Hospital, Cao County, Shandong, China

**Keywords:** fetal ovarian cyst, adnexal cyst, antenatal ultrasound, adnexal torsion, ovarian autoamputation, bacterial peritonitis, surgery

## Abstract

Intrauterine ovarian torsion with autoamputation (IOTA) in fetuses was uncommon. The vague and non-specific symptomatology of IOTA makes early diagnosis challenging. Potential complications, such as hemorrhagic infarction of the adnexal structures with the subsequent sequelae, may occur. Moreover, therapeutic options, such as conservative or surgical management, for IOTA remain uncertain in the literature. We report three cases of IOTA, two of which were complicated by peritoneal adhesion or spontaneous bacterial peritonitis, confirmed surgically and through laboratory studies. A suspected diagnosis of this uncommon condition was made preoperatively in two cases. Our case reports provided additional information about this rare condition, including the occurrence of complicated bacterial peritonitis, in neonates and infants with IOTA. A review of the literature on imaging diagnosis and management options for IOTA is also included.

## 1 Introduction

Although the exact incidence is not known, ovarian cysts in fetuses are estimated to occur in 1 in 1000 to 2,500 live births, largely due to the widespread use of prenatal ultrasound (US) screening and magnetic resonance imaging (MRI). The prenatal ovary cyst torsion rate is 10% (1, 2). Its pathogenesis may be associated with maternal hormone stimulation (3–5). Gonadotropins, maternal estrogens, and human chorionic gonadotropin from the placenta may influence the development of the fetal ovary and simple ovarian cysts. Studies have shown that the occurrence of fetal cysts is higher in pregnancies with maternal diabetes, toxemia, and Rh isoimmunization due to elevated levels of gonadotropins. Moreover, premature infants often have a connection with simple ovarian cysts, likely due to the underdevelopment of the hypothalamic–pituitary–ovarian feedback system (2). Anechoic cysts, described as “simple” cysts, may resolve spontaneously before birth or within the first several months of life (5, 6). However, adnexal torsion in a fetus, as a well-recognized complication of a larger adnexal cyst, can occur occasionally (7, 8). A complex heterogeneous fetal ovarian cyst, described as “complex,” usually suggests intracystic bleeding, septations, autoamputation, and dystrophic calcification. A solid component is a significant sonographic hallmark for the diagnosis (6, 9, 10).

Intrauterine adnexal autoamputation due to ovarian cysts is an extremely rare entity in the pediatric population. Autoamputation of the ovary cyst indicates a severe cyst pedicle torsion following total ischemia and necrosis of the adnexal tissues (11). Ovarian cyst torsion may be asymptomatic or present with non-specific symptoms. If the torsioned ovaries become autoamputated, they may be present as free-floating intraperitoneal masses (10, 12). Prenatal and postnatal ultrasound and MRI follow-up were very useful in detecting suspicious ovarian cysts with torsion or complex cysts (10). On prenatal ultrasound follow-up at an interval of 1 week, the appearance of a fluid-debris level is usually considered a notable indicator of ovarian cyst torsion (10). Autoamputated adnexa usually cannot resolve spontaneously and may cause complications, such as compression to the adjacent organ and tissue, peritoneal adhesion, adhesive small intestinal obstruction, and even bacterial peritonitis (3, 5, 8, 10, 13). The management options for prenatally detected ovarian cysts remain controversial (14). Prenatally diagnosed larger complex ovarian cysts are often managed surgically in an attempt to save the ovary (15). However, the management of prenatal adnexa torsion with adnexal autoamputation remains uncertain, including the timing of surgical intervention, conservative management, aspiration, and surgical approach (8, 14–20). Herein, we surgically treated three cases of intrauterine adnexal torsion with autoamputation (IOTA) and reviewed the literature to provide additional information on this rare disease entity.

## 2 Case reports

### 2.1 Case 1

**Figure 1:**
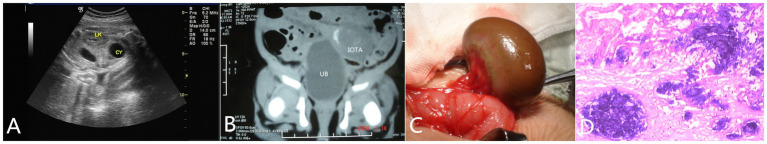
**(A)** Prenatal ultrasound scan showed a clear-cut cystic anechoic, round, unilocular, and thin-walled lesion, indicating a simple ovarian cyst originating from the left ovary. **(B)** Coronal CT image showed a solid mass with multiple calcifications. **(C)** Intraoperative findings showed a free-floating brownish lesion correlating to autoamputation of the left adnexa. **(D)** Histopathological examination showed tissue necrosis with focal calcification, with no sign of viable ovarian or fallopian tube tissue (H&E staining). LK, left kidney; CY, cyst; UB, urinary bladder; IOTA, intrauterine ovarian torsion with autoamputation.

A 45-day-old girl with a palpable abdominal mass was admitted to our clinic. A prenatal ultrasound screening detected an intra-abdominal cystic lesion of 1.62 cm × 1.22 cm × 1.60 cm at 28 weeks of gestation, which was a clear-cut cystic anechoic, round, unilocular, and thin-walled lesion, suggestive of a simple cyst of the left ovary (Figure 1A). No other anomalies were detected. The prenatal period had been uneventful. The baby was born vaginally at 39 weeks gestation, weighing 3,060 g. The infant was kept asymptomatic at birth. On admission, a mass of 3 cm in diameter was palpated in the left lower quadrant abdomen. Hematologic and biochemical investigations were normal. Tumor markers, including alpha-fetoprotein (α-AFP) and human chorionic gonadotropin (HCG), were within the normal range. The ultrasound follow-up at 6 weeks after birth showed a clear-cut solid lesion measuring 4.3 cm × 2.9 cm × 3 cm, containing hypoechoic and hyperechoic areas, suggestive of calcification in the left lower abdomen. An abdominal computerized tomography (CT) scan showed an uneven density mass of approximately 3.8 cm × 3 cm × 3 cm in the lower quadrant part of the abdomen, with an unclear boundary from the bladder wall. A strip-like high-density shadow was seen at the edge of the mass (Figure 1B).

At the age of 1.5 months, elective exploratory laparotomy via a left subumbilical transverse incision (mainly based on the location of the lesion) of 4 cm in length was performed. A free-floating, brownish, soft mass measuring 3.3 cm in diameter that attached to the greater omentum (Figure 1C) was identified in the left lower quadrant of the abdomen. The mass has a thin fibrous septum that is attached to the small intestine and posterior abdominal wall. The left ovary could not be identified with a blind ending of the left fallopian tube, indicating left ovarian autoamputation. The right ovary and fallopian tube looked normal, presented in their typical location, and were adequately suspended via the broad and ovarian ligaments. The autoamputated left ovarian mass was removed. Histopathological examination revealed a solid lesion of 5 cm × 4 cm × 3 cm with necrotic tissue and local calcification at the margin, without any viable ovarian tissue or signs of teratomatous elements or malignancy (Figure 1D). The postoperative recovery and 6-month follow-up were uneventful.

### 2.2 Case 2

A 2-day-old girl with a palpable abdominal mass was brought to our outpatient clinic. A prenatal ultrasound scan detected an intra-abdominal heterogeneous mass, 4 cm in diameter, at 35 weeks of gestational age. No other anomalies were detected. At 39^+3^ weeks of gestation, the baby was delivered by a cesarean section, weighing 3.55 kg. The infant remained asymptomatic. The physical examination revealed an abdominal mass, located in the right lower quadrant. Hematologic and biochemical parameters were normal except for a mildly elevated white blood cell count (12.51 × 10^9^/L, neutrophils, 55.3%). Two days after birth, an abdominal ultrasound follow-up revealed a clear-cut heterogeneous mass of 4.7 cm × 3.5 cm × 4.0 cm without internal blood flow, suggestive of ovarian torsion. Exploratory laparotomy via a right paraumbilical vertical incision was performed. Intraoperative findings revealed that there was a small amount of peritoneal effusion that was collected for bacterial culture. A free-floating brownish solid mass was identified in the right lower quadrant of the abdomen, attached to sigmoid colon mesentery with thin fibrous septa. The right ovary could not be identified, and the right fallopian tube was a blind end, indicating autoamputation of the right adnexa. The uterus, the left ovary, and the fallopian tube looked normal. The autoamputated ovary was removed after aspiration of intracystic dark-red fluid (10 mL). *Staphylococcus aureus* was positive in the ascitic fluid culture and was sensitive to ceftazidime in antimicrobial susceptibility testing. A diagnosis of prenatal adnexal torsion with autoamputation complicated by bacterial peritonitis was confirmed. The baby girl received a 5-day course of ceftazidime (25 mg/kg intravenously, b.i.d.) postoperatively.

The histopathological examination revealed the mass contained connective tissue and calcification, without viable ovarian tissue, teratomatous elements, or malignancy, suggesting antenatal adnexal torsion. A paratubal cyst of 3 mm in diameter with a stalk in the mesentery of the left fallopian tube was removed, and the pathological examination showed an embryonic cyst. The postoperative recovery and 3-month follow-up were uneventful.

### 2.3 Case 3

A 6-week-old girl was transferred to our clinic with a palpable abdominal mass. A prenatal ultrasound scan found an intra-abdominal heterogeneous cystic mass at 35 weeks of gestational age. No other anomalies were detected. At 39 weeks of gestation, the baby was delivered by cesarean section, weighing 3,200 g. The infant remained asymptomatic. On admission, the physical examination revealed an abdominal mass of 7 cm in diameter in the right lower quadrant. Hematologic and biochemical parameters were normal. Ultrasound showed that a heterogeneous mass with focal hyperechoic areas on the margin, 7.5 cm × 5.4 cm × 3.9 cm in size, was located in the pelvis, suggestive of an ovarian cyst torsion with autoamputation and calcification. Laparoscopic exploration was performed via a 5-mm port that was inserted in the umbilical position for a 30° camera and two 3-mm ports that were inserted in the right or left mid-abdomen for surgical instruments, respectively. Laparoscopic exploration showed a free-floating brownish cystic mass located in the pelvis and the right lower quadrant, without an adhesion band. The right adnexa was absent. The left ovary and the fallopian tube appeared normal. The right ovarian autoamputation was confirmed and excised completely.

The histopathological examination revealed the mass containing necrotic tissues and scattered calcification, without viable ovarian tissue.

The postoperative period was uneventful, and the infant was discharged home 6 days after surgery.

## 3 Discussion

The incidence of fetal abdominal cysts detected on prenatal US scans, including a variety of clinical entities, has been increasing due to improved imaging screening (4, 13, 21–23). Approximately 63% of female fetuses detected with intra-abdominal cysts on prenatal US scans are of ovarian origin. It is reported that approximately 30% of female neonates have ovarian cysts (22). Prenatal adnexal torsion will result in autoamputation due to hemorrhagic infarction, as seen in our cases. They are considered not to resolve spontaneously and cause clinical diagnostic uncertainty (5, 24). Our second and third cases presented a complex cyst on postnatal ultrasound check, suggestive of antenatal ovarian torsion with adnexal autoamputation.

Regarding the image appearance, based on pathological findings, fetal and neonatal ovarian cysts are usually classified as simple or complex on an ultrasound scan; the former is thin-walled, anechoic, homogeneous, and unilocular, whereas the latter is often thick-walled with a solid structure and septa and fluid-debris level (25).

Fetal ovarian cyst torsion may occur in the perinatal period. A complex cyst with the fluid-debris level, solid components, and calcification on ultrasound examination should be considered a significant hallmark of adnexal torsion and autoamputation (4, 5, 10). An MRI can reveal a complex cyst with a fluid level, and it can also be used in patients with an equivocal diagnosis or suspicion of malignancy (10). The CT scan can show a low-density mass with calcification (14, 26), as seen in our first case. The MRI and CT scans may enhance diagnostic accuracy (5).

Antenatal ovarian torsion (AOT) may mimic other intra-abdominal malformations, such as gastrointestinal duplication cysts, lymphatic malformations, mesenteric cysts, serous cystadenomas, urachal cysts, simple hepatic cysts, meconium pseudocysts, and retroperitoneal cystic teratomas (27–30). Making a precise prenatal diagnosis is difficult; an early ultrasound scan after birth is needed for differential diagnosis (31, 32). In our case reports, IOTA was diagnosed before surgical intervention and was confirmed in the latter two cases.

In all, ultrasound scans are usually used for the diagnosis of fetal or neonatal intraabdominal lesions (3, 33–35). Tyraskis et al. (13) recently concluded that fluid-debris levels, solid components, and calcifications on ultrasound scans may indicate ovarian loss caused by previous severe torsion. While for those with an ambiguous diagnosis of IOTA, MRI and CT scans may enhance diagnostic accuracy (36–38).

Regarding the complications of the fetal ovarian cysts, the larger cysts (>4.0 cm in diameter) will increase the occurrence of complications, including torsion, rupture, intracystic bleeding, bacterial peritonitis, compression, and adhesive intestinal obstruction (13, 19, 22, 27, 39, 40). In our case series, intraoperative findings revealed that autoamputated adnexa had an attachment to the posterior peritoneum, small intestine, or sigmoid mesentery with thin fibrous septa, which pose a risk of long-term complication of peritoneal adhesion, even adhesive intestinal obstruction (41). It is assumed that inflammation and subsequent adhesions were caused by a torsed necrotic ovary (2). Our first case had a simple cyst first detected by an ultrasound scan at 28 weeks of gestational age. Postnatal ultrasound scan showed the lesion becoming a clear-cut solid mass containing hypoechoic and hyperechoic areas, suggestive of IOTA; the second and third cases represented a cystic lesion with anechoic and fibrous echoes at 38 and 35 weeks of gestation, respectively, indicating IOTA. Moreover, our second case presented with bacterial peritonitis, which has not been reported before. As for the complication of peritonitis, it is seldom reported in the literature. Nichols and Julian (40) described the occurrence of infection and peritonitis followed by adnexal torsion with ovary gangrene. In our case reports, a 2-day-old girl had *Staphylococcus aureus* growth in abdominal effusion. This may be associated with gangrenous cysts and needs to be further investigated (25, 40).

Treatment options for neonates and infants with antenatally detected ovarian cysts included conservative management, image-guided percutaneous aspiration, laparoscopy-guided aspiration, laparoscopic or open cystectomy, or oophorectomy. Until now, the evidence on the treatment of fetal ovarian cysts is very limited (1).

The decision to intervene should depend on cyst size, ultrasound characteristics, lesion progression, and clinical representation (10, 18, 21, 22, 27, 33, 34, 39).

Most simple cysts of <4 cm in diameter and approximately half of complex cysts will resolve spontaneously before birth and within 6 months of age (4, 15, 16, 42). Data have shown that there is no advantage for anechoic fetal cysts via prenatal aspiration or in-utero aspiration (IUA), so it should be performed individually (31, 41). However, it is reported that ultrasound-guided prenatal aspiration of simple ovarian cysts >40 mm seems to be related to a significant benefit in terms of preservation of the ovarian parenchyma (1). These risks must be weighed against the probability of cyst regression (31). However, compared to expectant management, IUA was associated with a reduced oophorectomy rate (43). Surgical removal should be considered when the cyst rapidly increases in size, develops complications, or fails to resolve (4, 44). Conservative versus surgical treatment for IOTA on postnatal ultrasound scans remains controversial. Postnatal observation, advocated as initial management, regardless of cyst size or ultrasound characteristics, spares 50% of newborn babies with prenatal ovarian cyst torsion from unnecessary surgical procedures and does not increase oophorectomy rate (16, 21, 27, 36, 39). We choose surgical intervention for IOTA due to the symptom of a palpable abdominal mass. Table 1 summarizes the management options according to guidelines and literature (1, 2, 21, 42, 45–50).

**Table 1:** The management options for ovarian cysts in fetuses and infants.

USS appearance	Simple		Complex	
	DIA <4 cm	DIA ≥4 cm		
Prenatal	USS follow-up, per 7 days	USS-guided aspiration	USS follow-up, per 3–6 weeks	
Postnatal	USS follow-up per 3–6 weeks until the cyst resolved spontaneously	USS or laparoscopy-guided cyst aspiration or fenestration	DIA ≥4 cm → surgery: Laparoscopic; Video-assisted; Trans-umbilical	Cyst unroofing, cystectomy; autoamputated ovary excision
	*USS appearance becoming complex; symptomatic cysts; cyst persistence >6 months → surgical intervention			
			*Observation and follow-up	

DIA, diameter; USS, ultrasound scan.

Regarding the timing of surgical intervention, it is difficult to manage in the early days of life, and early surgical intervention will not improve ovarian preservation (2, 13). Whether an early surgical intervention can improve the outcome in those neonates with ovarian cyst torsion detected prenatally is yet to be established. Neonates with large ovarian cysts (>40 mm) or cysts with fluid-debris levels (the intracystic hemorrhage appearance of an ultrasound scan) usually require early surgical treatment to save the viable ovarian tissue (3, 4, 36, 51, 52). However, once adnexal autoamputation occurs prenatally, the ovary usually is no longer salvageable, and surgical intervention in neonates without symptoms may add risk with no benefit except making the diagnosis (36).

Autoamputated ovarian can be safely excised due to no viable ovary tissue (8, 10). Surgical intervention, such as laparoscopic cyst aspiration and fenestration, can be given for simple ovarian cysts >4 cm within the 2 weeks of life, and serial ultrasound follow-up should last 3 months (40). For a torsioned necrotic-appearing ovarian cyst, viable ovarian tissue should be preserved wherever possible (18, 40).

In conclusion, ovarian cyst torsion with autoamputation in a fetus was uncommon. We reported three patients with IOTA, including one patient complicated with peritoneal adhesion and another patient with bacterial peritonitis. Surgical exploration, including a laparoscopic approach and an excision of the autoamputated ovarian cysts, was needed for each patient with a good outcome. Further research is needed to better define the management options of IOTA, and a guideline is deemed essential to avoid unnecessary surgical interventions.

## Data Availability

The raw data supporting the conclusions of this article will be made available by the authors, without undue reservation.

## References

[1] Chiarenza SF Conighi ML Conforti A Bleve C Esposito C Escolino M. Guidelines of the Italian Society of Video surgery in infancy (SIVI) for the minimally invasive treatment of fetal and neonatal ovarian cysts. Pediatr Med Chir. (2020) 42:242. doi: 10.4081/pmc.2020.242, PMID: 33140631

[2] Saxena AK Mutanen A Gorter R Conforti A Bagolan P De Coppi P. European Paediatric Surgeons' Association. European Paediatric Surgeons' Association consensus statement on the management of neonatal ovarian simple cysts. Eur J Pediatr Surg. (2024) 34:215–21. doi: 10.1055/s-0043-1771211, PMID: 37557903

[3] Rotar IC Tudorache S Staicu A Popa-Stanila R Constantin R Surcel M. Fetal ovarian cysts: prenatal diagnosis using ultrasound and MRI, management and postnatal outcome-our centers experience. Diagnostics. (2021) 12:89. doi: 10.3390/diagnostics12010089, PMID: 35054256 PMC8775004

[4] Zampieri N Scirè G Zambon C Ottolenghi A Camoglio FS. Unusual presentation of antenatal ovarian torsion: free-floating abdominal cysts. Our experience and surgical management. J Laparoendosc Adv Surg Tech A. (2009) 19:S149–52. doi: 10.1089/lap.2008.0128.supp, PMID: 18973466

[5] Tseng D Curran TJ Silen ML. Minimally invasive management of the prenatally torsioned ovarian cyst. J Pediatr Surg. (2002) 37:1467–9. doi: 10.1053/jpsu.2002.35415, PMID: 12378456

[6] Romiti A Moro F Ricci L Codeca C Pozzati F Viggiano M. Using IOTA terminology to evaluate fetal ovarian cysts: analysis of 51 cysts over 10-year period. Ultrasound Obstet Gynecol. (2023) 61:408–14. doi: 10.1002/uog.26061, PMID: 36123819

[7] Parlak A Celik F Turedi Sezer B Yilmaz MU Kilic N Kiristioglu I. Laparoscopy is a definitive diagnostic method for auto-amputated ovary in infants. Pediatr Surg Int. (2022) 38:1649–55. doi: 10.1007/s00383-022-05192-1, PMID: 35964259

[8] Ladenhauf HN Brandtner MG Ardelean MA Schimke C Metzger R. Laparoscopic management of autoamputated ovary in newborns: a report of 2 cases. J Minim Invasive Gynecol. (2017) 24:859–62. doi: 10.1016/j.jmig.2017.04.006, PMID: 28450253

[9] Muto M Yoshizato T Horinouchi T Yokomine M Sakamoto Y Ishii S. Risk factors in fetal ovarian cysts for postnatal adverse outcomes. Kurume Med J. (2024) 69:127–33. doi: 10.2739/kurumemedj.MS6934002, PMID: 38233187

[10] Ozcan HN Balci S Ekinci S Gunes A Oguz B Ciftci AO. Imaging findings of fetal-neonatal ovarian cysts complicated with ovarian torsion and autoamputation. AJR Am J Roentgenol. (2015) 205:185–9. doi: 10.2214/AJR.14.13426, PMID: 26102397

[11] Koike Y Inoue M Uchida K Kawamoto A Yasuda H Okugawa Y. Ovarian autoamputation in a neonate: a case report with literature review. Pediatr Surg Int. (2009) 25:655–8. doi: 10.1007/s00383-009-2396-9, PMID: 19513725

[12] Pal S Kumari P Jain A Sinha SK. Fetal ovarian cyst managed laparoscopically in the neonatal period. Indian Pediatr. (2020) 57:866–7. doi: 10.1007/s13312-020-1974-8, PMID: 32999123

[13] Tyraskis A Davidson J Billington J Blackburn S Curry J Mullassery D. Ultrasonographic features associated with previous torsion and the impact of surgery in managing neonatal ovarian cysts: a 20-year single-Centre retrospective study. Pediatr Surg Int. (2023) 39:185. doi: 10.1007/s00383-023-05458-2, PMID: 37095416 PMC10125918

[14] Enríquez G Durán C Torán N Piqueras J Gratacós E Aso C. Conservative versus surgical treatment for complex neonatal ovarian cysts: outcomes study. AJR Am J Roentgenol. (2005) 185:501–8. doi: 10.2214/ajr.185.2.01850501 PMID: 16037528

[15] Rialon KL Akinkuotu A Fahy AS Shelmerdine S Traubici J Chiu P. Management of ovarian lesions diagnosed during infancy. J Pediatr Surg. (2019) 54:955–8. doi: 10.1016/j.jpedsurg.2019.01.027, PMID: 30795909

[16] Safa N Yanchar N Puligandla P Sewitch M Baird R Beaunoyer M. And Canadian consortium for research in pediatric surgery (can CORPS). Treatment and outcomes of congenital ovarian cysts a study by the Canadian consortium for research in pediatric surgery (can CORPS). Ann Surg. (2023) 277:e1130–7. doi: 10.1097/SLA.0000000000005409, PMID: 35166261 PMC10082055

[17] Tyraskis A Bakalis S David AL Eaton S De Coppi P. A systematic review and meta-analysis on fetal ovarian cysts: impact of size, appearance and prenatal aspiration. Prenat Diagn. (2017) 37:951–8. doi: 10.1002/pd.5143, PMID: 28886226

[18] Hara T Mimura K Endo M Fujii M Matsuyama T Yagi K. Diagnosis, management, and therapy of fetal ovarian cysts detected by prenatal ultrasonography: a report of 36 cases and literature review. Diagnostics. (2021) 11:2224. doi: 10.3390/diagnostics11122224, PMID: 34943461 PMC8700714

[19] López Soto Á Bueno González M Urbano Reyes M Carlos Moya Jiménez L Beltrán Sánchez A Garví Morcillo J. Imaging in fetal genital anomalies. Eur J Obstet Gynecol Reprod Biol. (2023) 283:13–24. doi: 10.1016/j.ejogrb.2023.01.035, PMID: 36750003

[20] Zhou JL Zhu XC Fang YL Huang R Wang QY Ge WP. Bilateral fetal ovarian autoamputation. Arch Dis Child Fetal Neonatal Ed. (2021) 106:204. doi: 10.1136/archdischild-2020-318902, PMID: 32546541

[21] Papic JC Billmire DF Rescorla FJ Finnell SM Leys CM. Management of neonatal ovarian cysts and its effect on ovarian preservation. J Pediatr Surg. (2014) 49:990–4. doi: 10.1016/j.jpedsurg.2014.01.040, PMID: 24888849

[22] Bryant AE Laufer MR. Fetal ovarian cysts: incidence, diagnosis and management. J Reprod Med. (2004) 49:329–37. PMID: 15214704

[23] Sanna E Loukogeorgakis S Prior T Derwig I Paramasivam G Choudhry M. Fetal abdominal cysts: antenatal course and postnatal outcomes. J Perinat Med. (2019) 47:418–21. doi: 10.1515/jpm-2018-0311, PMID: 30763268

[24] Visnjic S Domljan M Zupancic B. Two-port laparoscopic management of an autoamputated ovarian cyst in a newborn. J Minim Invasive Gynecol. (2008) 15:366–9. doi: 10.1016/j.jmig.2007.11.001, PMID: 18439514

[25] Nussbaum AR Sanders RC Hartman DS Dudgeon DL Parmley TH. Neonatal ovarian cysts: sonographic-pathologic correlation. Radiology. (1988) 168:817–21. doi: 10.1148/radiology.168.3.3043551, PMID: 3043551

[26] Meyer JS Harmon CM Harty MP Markowitz RI Hubbard AM Bellah RD. Ovarian torsion: clinical and imaging presentation in children. J Pediatr Surg. (1995) 30:1433–6. doi: 10.1016/0022-3468(95)90399-2, PMID: 8786481

[27] Akalin M Demirci O Dayan E Odacilar AS Ocal A Celayir A. Natural history of fetal ovarian cysts in the prenatal and postnatal periods. J Clin Ultrasound. (2021) 49:822–7. doi: 10.1002/jcu.23044, PMID: 34245032

[28] Kim HS Yoo SY Cha MJ Kim JH Jeon TY Kim WK. Diagnosis of neonatal ovarian torsion: emphasis on prenatal and postnatal sonographic findings. J Clin Ultrasound. (2016) 44:290–7. doi: 10.1002/jcu.22327, PMID: 27154434

[29] Cass DL. Fetal abdominal tumors and cysts. Transl Pediatr. (2021) 10:1530–41. doi: 10.21037/tp-20-440, PMID: 34189111 PMC8192983

[30] Lee W Lee MY Teo H. Ultrasound and alternative multimodality imaging of intra-abdominal and pelvic cystic masses in the newborn. Ultrasound. (2021) 29:241–51. doi: 10.1177/1742271X20984814, PMID: 34777544 PMC8579366

[31] Heling KS Chaoui R Kirchmair F Stadie S Bollmann R. Fetal ovarian cysts: prenatal diagnosis, management and postnatal outcome. Ultrasound Obstet Gynecol. (2002) 20:47–50. doi: 10.1046/j.1469-0705.2002.00725.x, PMID: 12100417

[32] Toker Kurtmen B Divarci E Ergun O Ozok G Celik A. The role of surgery in antenatal ovarian torsion: retrospective evaluation of 28 cases and review of the literature. J Pediatr Adolesc Gynecol. (2022) 35:18–22. doi: 10.1016/j.jpag.2021.08.007, PMID: 34454073

[33] Safa N Yanchar N Puligandla P Sewitch M Baird R Beaunoyer M. The Canadian consortium for research in pediatric surgery can CORPS. Differentiating congenital ovarian cysts from other abdominal cystic lesions in female infants: a study by the Canadian consortium for research in pediatric surgery (can CORPS). J Pediatr Surg. (2022) 57:877–82. doi: 10.1016/j.jpedsurg.2021.12.043, PMID: 35090716

[34] Chen L Hu Y Hu C Wen H. Prenatal evaluation and postnatal outcomes of fetal ovarian cysts. Prenat Diagn. (2020) 40:1258–64. doi: 10.1002/pd.5754, PMID: 32441348

[35] Hadian F Rutten C Siddiqui I Tomlinson C Chavhan GB. Neonatal liver imaging: techniques, role of imaging, and indications. Radiographics. (2024) 44:e240034. doi: 10.1148/rg.240034, PMID: 39509288

[36] Trotman GE Zamora M Gomez-Lobo V. Non-surgical management of the auto-amputated adnexa in the neonate: a report on two cases. J Pediatr Adolesc Gynecol. (2014) 27:107–10. doi: 10.1016/j.jpag.2013.06.019, PMID: 24075090

[37] Ros PR Olmsted WW Moser RP Jr Dachman AH Hjermstad BH Sobin LH. Mesenteric and omental cysts: histologic classification with imaging correlation. Radiology. (1987) 164:327–32. doi: 10.1148/radiology.164.2.3299483, PMID: 3299483

[38] Karavadara D Davidson JR Story L Diab Y Upadhyaya M. Missed opportunities for ovarian salvage in children: an 8-year review of surgically managed ovarian lesions at a tertiary pediatric surgery Centre. Pediatr Surg Int. (2021) 37:1281–6. doi: 10.1007/s00383-021-04935-w, PMID: 34235545 PMC8325645

[39] Bascietto F Liberati M Marrone L Khalil A Pagani G Gustapane S. Outcome of fetal ovarian cysts diagnosed on prenatal ultrasound examination: systematic review and meta-analysis. Ultrasound Obstet Gynecol. (2017) 50:20–31. doi: 10.1002/uog.16002, PMID: 27325566

[40] Nichols DH Julian PJ. Torsion of the adnexa. Clin Obstet Gynecol. (1985) 28:375–80. doi: 10.1097/00003081-198528020-00015, PMID: 4017325

[41] Karakuş OZ Ateş O Hakgüder G Olguner M Akgür FM. Complex fetal ovarian cysts cause problems even after regression. Eur J Pediatr Surg. (2014) 24:337–40. doi: 10.1055/s-0033-1348023, PMID: 23737131

[42] Focseneanu MA Omurtag K Ratts VS Merritt DF. The auto-amputated adnexa: a review of findings in a pediatric population. J Pediatr Adolesc Gynecol. (2013) 26:305–13. doi: 10.1016/j.jpag.2012.08.012, PMID: 23287601

[43] Diguisto C Winer N Benoist G Laurichesse-Delmas H Potin J Binet A. In-utero aspiration vs. expectant management of anechoic fetal ovarian cysts: open randomized controlled trial. Ultrasound Obstet Gynecol. (2018) 52:159–64. doi: 10.1002/uog.18973, PMID: 29205608

[44] Lewis S Walker J McHoney M. Antenatally detected abdominal cyst: does cyst size and nature determine postnatal symptoms and outcome? Early Hum Dev. (2020) 147:105102. doi: 10.1016/j.earlhumdev.2020.105102, PMID: 32521469

[45] Akın MA Akın L Özbek S Tireli G Kavuncuoğlu S Sander S. Fetal-neonatal ovarian cysts--their monitoring and management: retrospective evaluation of 20 cases and review of the literature. J Clin Res Pediatr Endocrinol. (2010) 2:28–33. doi: 10.4274/jcrpe.v2i1.28, PMID: 21274333 PMC3005663

[46] Puligandla PS Laberge JM. Lethal outcome after percutaneous aspiration of a presumed ovarian cyst in a neonate. Semin Pediatr Surg. (2009) 18:119–21. doi: 10.1053/j.sempedsurg.2009.02.012, PMID: 19349004

[47] Schenkman L Weiner TM Phillips JD. Evolution of the surgical management of neonatal ovarian cysts: laparoscopic-assisted transumbilical extracorporeal ovarian cystectomy (LATEC). J Laparoendosc Adv Surg Tech A. (2008) 18:635–40. doi: 10.1089/lap.2007.0193, PMID: 18721022 PMC3198621

[48] Shapiro EY Kaye JD Palmer LS. Laparoscopic ovarian cystectomy in children. Urology. (2009) 73:526–8. doi: 10.1016/j.urology.2008.08.497, PMID: 19038433

[49] Anders JF Powell EC. Urgency of evaluation and outcome of acute ovarian torsion in pediatric patients. Arch Pediatr Adolesc Med. (2005) 159:532–5. doi: 10.1001/archpedi.159.6.532, PMID: 15939851

[50] Zampieri N Mantovani A Scirè G Camoglio FS. Neonatal surgery for giant floating abdominal cysts in females: clinical and surgical management. J Pediatr Adolesc Gynecol. (2014) 27:271–3. doi: 10.1016/j.jpag.2013.11.012, PMID: 24841518

[51] Ogul H Havan N Pirimoglu B Guvendi B Kisaoglu A Kantarci M. Prenatal and postnatal ultrasonographic findings of the torsioned ovarian cyst: a case report and brief literature review. Int Surg. (2015) 100:514–7. doi: 10.9738/INTSURG-D-14-00005.1, PMID: 25785337 PMC4370545

[52] Galinier P Carfagna L Juricic M Lemasson F Moscovici J Guitard J. Fetal ovarian cysts management and ovarian prognosis: a report of 82 cases. J Pediatr Surg. (2008) 43:2004–9. doi: 10.1016/j.jpedsurg.2008.02.060, PMID: 18970932

